# Book Reviews

**Published:** 1843-06

**Authors:** 


### Bibliographical Notices.

*The British Quarterly Journal of Dental Surgery.* Edited by J. Robinson, Esq. London; John Churchill, Princes street, Soho; Otis Clapp, School street, Boston; vol. 1, No. 1, pp. 64.

It is with something more than fraternal feelings that we hail the appearance of the March number, the first of the series, of the British Quarterly Journal of Dental Surgery. If we have not quite the feelings of paternity, we certainly have those of an elder brother, who salutes, in the new accession to the family of a common parent, a future compeer in the fields of thrifty industry, a friend and co-labourer in the pursuits of science and achievement, as connected with an arduous and indispensable department of organic surgery. Although science, like universal philanthropy, is the exclusive growth and production of no one country, yet national pride is somewhat gratified to find either our own country, or that from which we boast our derivation, among the foremost in the productions of mind and genius. The United States of America have always been loyal to British talent, science and literature. No revolution has ever broken us off as a fragment from the mighty empire of the English language and English intellectuality. The empire of letters ever was a republic, and no severance from a monarchical form of government could affect literary and scientific relations among the peers of philosophy. Our Franklin, Jefferson, Rush and Physic were as much the magnates of the Old England as of the New; they belonged to the English language and to philosophy; and their imperishable memorials add to the wealth of English mind, which has been in the progress of accumulation for a thousand years; and the grateful consideration that the American Journal and Library of Dental Science is three years older than its transatlantic brother, we receive rather in the light of a good old English endorsement on the back of our own paper, calculated to make it more marketable and of higher value in the mart of mind, where the rate of scientific exchange has often been against a new country and in favour of the old.

With the light of three years' experience diffused around our path; with moral and scientific benefits returned into our own bosoms a thouand-fold for all the sacrifices and exertions we have made for the advancement of dental surgery; with the three-fold power of a National Society of Dentists, a College of Dental Surgery, and a Journal of Dentistry, in full and successful operation; and with hundreds of facts attesting the rapid rise of our profession into eminence, respectability, and scientific achievement;—we cordially reach our hand, in fraternal grasp, across the only barrier that can divide English from American science—a barrier which that very science has narrowed down to a pleasure passage of two weeks, scarcely interrupting the pulsations of intellect, throbbing through the mental Anglo-Saxon system—and we bid our young brother, over the seas, God speed, with our heart's benison.

The Introductory Address is well written, and contains this expression of good feeling and amity.

"And yet we will confess that our brethren in the United States have been beforehand with us; our Journal is but a younger brother of the admirable periodical published in America. In this, as in all other things, our brethren have 'gone ahead;' we shall again and again have to refer to, and avail ourselves of their useful labours; and we feel certain the relations between us and them will continue, as our feelings now are, to be truly fraternal."

As one of the ultimate objects of the Journal is declared "to be the instrument of so uniting our present scattered profession, as that it shall come before the government of the country, with a claim grounded in the strength of right, to be incorporated, and to have a power of instituting examinations, and granting diplomas to worthy candidates," we certainly feel more than a common sympathy with this desirable event, knowing as we do, how much the prosperity of the periodical will be promoted by the establishment of a National Society of Dental Surgeons. We were quite astonished to find, in the editorial remarks and in those of the various correspondents, so many coincidences between the state of professional dentistry in England, and that of this country at the time of the formation of our National Association. We had thought that, in the older countries, this important branch of surgery had been more guarded from the obtrusions of empiricism. But, as "a beginning is always more than half the battle," we anticipate from this movement, in the great metropolis of Britain, consequences quite as salutary to this department of the curative art in England, as those we now witness in the progress of development in this country.

We also learn, from a well written editorial in the Quarterly, that the subject of the formation of an association of dental surgeons had been "dined" upon in London. The fact of the dining indicates good appetite, and the antecedent circumstance of the dental gentlemen having good teeth, either natural or acquired; but this alone furnishes no evidence of the ability of the diners to make good teeth for others.

Should a dental association be formed, there will be much labour created for other organs—for hand, head and heart—and many sacrifices of ease and pleasure must be the consequence; to be rewarded, however, in substantial consideration among the educated and leading classes of the community, and in general and moral elevation.

We sincerely hope that the noble undertaking of the formation of a National Association of Dental Surgeons, in England, will not be abandoned. Such an organization, if properly formed and conducted, would contribute both to the advancement of dental science and the elevation of the profession in that country. Meanwhile, we tender, to those who are engaged in this praiseworthy undertaking, our best wishes for their success.

The British Quarterly has honoured one of the editors of this Journal, Dr. Solyman Brown, by commencing the republication of his "Treatise on Mechanical Dentistry," which has appeared in our Journal. With regard to the merits of this treatise, the editor thus remarks:—

"This treatise contains the best practical information upon a subject intimately connected with the objects of our Journal, and it is our intention to lay before our readers the whole series of the work, distinguished as it is by lucid and distinct views, and much and various erudition. Our author modestly professes no higher purpose than that of instructing young men, on their first entry into the profession, and developing certain general and fundamental principles, from phenomena constantly recurring. We cannot too much commend the method of his treatise, believing, as we do most heartily, that nothing is more likely to communicate a habit of observation, or to strengthen the memory of facts once acquired, than a perpetual endeavour to refer to their true causes, effects which are continually passing before us.

"The merit of these papers, as we before observed, consists in their clearness and precision, qualities which particularly adapt them to the wants of the student.

"The author has separated from the results of mere conjecture, those conclusions which may be safely relied on as furnishing maxims and directions for progressive improvement in mechanical dentistry. He also marks, perspicuously, the circumstances that most frequently accompany the total failure of that branch of the art. His unassuming manner adds an additional value to his labours, and we should be sorry to be insensible to such an appeal as the following, even upon occasions calling for less of acknowledgment and approbation."

We know that there are many men of a very high order of talent in the profession in England, and we hope that they, with one accord, will contribute promptly, both with their pens and their money, to the support of this, the first periodical, devoted exclusively to the science of dental surgery, ever published in Europe. We also trust that the profession in this country will extend to it their aid. H.

*A Dictionary of Practical Surgery, &c. &c.* By SAMUEL COOPER, senior surgeon to the University College Hospital, &c. &c. With numerous notes and additions, &c. together with a supplementary index, in which the science of surgery is brought down to the present period, including all recent improvements in Europe and America. By DAVID MEREDITH REESE, A. M., M. D., &c. &c. NEW YORK, HARPER & BROTHERS, pp. 1164, 1842.

Professor Reese, now of the Washington University of Baltimore, has furnished the medical profession with a new and improved edition of that standard work, the Dictionary of Surgery, by Samuel Cooper.

In addition to the numerous notes upon American surgery, which were found in the former reprint from the pen of Professor Reese, all of which have been adopted by Mr. Cooper in his latest edition, the American editor has now added a supplementary appendix, which embodies every recent improvement in the science, whether at home or abroad, including all the new operations and splendid achievements of the surgeons of the United States, particularly in orthopædic and autoplastic surgery.

The republication of the numerous contributions of Professor Reese to this dictionary, in Europe, as has been heretofore done by Mr. Cooper in London, and M. Velpeau in France, and will now be again done in their forthcoming volumes, cannot fail to exalt the merits of our countrymen in this department, to which patriotic work the American editor has devoted himself with great zeal and industry. More than a hundred of our American surgeons, in different parts of our country, are named here in connection with their surgical claims, which are set forth with impartiality and justice. We are gratified to find that the labours of Professor Reese have been appreciated by his professional brethren at home and abroad, and that the recent edition has called forth the warmest testimonials of approval from such men as Mott, Stevens, Parker, Detmold and Francis, of New York; Gibson, McClellan, and Mutter, of Philadelphia, and Smith, Dunbar and Baxley, of Baltimore. We need scarcely add, that Professor Reese's edition of Cooper's

Surgical Dictionary is the text book in every medical college in the country; the venerable Cooper himself having given it his cordial approbation, in testimonials to its merits which will be found in the work. H.

*Observations on the Extraction of Teeth, with plates.* By J. Chitty Clendon, Surgeon Dentist, London. Highley, Fleet street, 1843.

Of the merits of the above named work, we are not able to speak, not having yet seen it. There is, however, a short review of it in the "British Quarterly Journal of Dental Surgery," and from which we quote the following:—

"In conclusion we approve highly of the general aim of Mr. Clendon's work, and without undertaking to defend all his opinions, we recommend the book strongly to our readers, as well deserving a place in the library of every practitioner and student. It possesses the unusual merit of clear and concise language; and is neither so technical nor so extended as to prove tedious to the general reader."

